# Strategies for Implementing Building Operability Certificate to Improve Performance of Building Management: A Case Study in Probolinggo City, Indonesia

**DOI:** 10.1155/2024/8749628

**Published:** 2024-01-06

**Authors:** Dwi Putranto Riau, Rulinawaty, Dwi Siswahyudi, Muhtarom

**Affiliations:** ^1^Graduate School, Universitas Terbuka, Jakarta, Indonesia; ^2^Department of Civil Engineering, Faculty of Engineering, Universitas Negeri Malang, Malang, Indonesia; ^3^Department of Public Administration, Faculty of Law, Social and Political Sciences, Universitas Terbuka, Jakarta, Indonesia

## Abstract

An operability certificate is required before using a building. The building operability certificate concerns occupants' safety before the building is used and is directed by Law Government Regulation 16 of 2021 for building security. This article discusses implementing a building operability certificate underperforming and the strategy for implementing the building operability certificate (SLF) policy. This research uses a descriptive qualitative method. First, qualitative data analysis utilizing SWOT determined the building operability certificate implementation strategy. Second, ranking strategies are analyzed to identify (QSPM's) priorities. According to the regulation that every building must have SLF, the building operability certificate was not appropriately implemented. With the current building information system legislation, each region can issue Building Approval (PBG) and building operability certificate (SLF). Research findings based on SWOT and QSPM are used to generate seven strategies: Regional Apparatus Work Units (SKPD) commitment, increased socialization, regulation implementation, capacity building, optimal communication, SIMBG implementation, and increasing resources.

## 1. Introduction

In places where the building operability certificate (SLF) implementation is subject to different performance limits that are not in line with the rules' aims, the building operability certificate (SLF)-implemented districts face these limits [1]. Probolinggo City, which has implemented SLF since 2008, has not followed local regulations when implementing the building operability certificate 2008–2019: 45 SLF issued. Investment Board and One-Stop Licensing Services issue 400 IMBs per year. The functional worthiness certificate is just 5 per year [2, 3]. The performance of SKPD buildings that could have been more optimal in implementing and issuing SLF is required to find a solution by paying attention to and continuing to improve human resources, communication, bureaucratic structure, disposition, and external factors starting from public support and legislative support.

Several policy models utilize top-down policies in policy implementation, including George Edward III's success and failure factor method. Four factors contribute to successful implementation. Communication, resources, disposition, and bureaucratic structure affect policy execution [4].

According to Edward III, policy decisions and instructions must be applied to the right people and communicated to people who are clear and accurate so that they can be understood quickly by the executor. Effective policy implementation according to Edward III (1984) is that policy implementers must know what they have to do, policy decisions must be transmitted (transmission) to the right people so that the communication must be accurately received clearly (clarity), and also order policies must be consistent [5, 6].

Stoner et al. (1997: 47) argued that in implementing a program to successfully achieve policy objectives, the bureaucratic structure factor holds an important position. In this bureaucratic structure, there are divisions of tasks and functions as well as a delegation of authority. With the delegation of authority, the organization can function efficiently [7, 8].

The bureaucratic organization or building organizer that handles the building operability certificate (SLF) since 2008 in the field of Urban Arrangement and Building Supervision has changed along with the institutional structure of the Probolinggo City Public Works Department. In 2012, the Cipta Karya and Spatial Planning sector implemented two sections: the Spatial Planning section and the Building and Environmental Structuring Section, which handled building permit recommendations [9].

Edwards III (1980: 11) noted that effective implementation requires staff of the right size and expertise, relevant and adequate information on how to implement policies, and on the compliance of others involved in implementation, the authority to ensure that policies are carried out as intended, and facilities (including buildings, land, supplies, and equipment) [5].

The apparatus resources that handle building implementation, especially the Building Approval (PBG) recommendation and the building operability certificate, are inadequate. Six technical and nontechnical devices handle the building operability certificate (SLF). Existing staff in building management demands an improvement in HR competences, for example, in the Building Approval (PBG) and building operability certificate (SLF) services through SIMBG [10, 11], which is one way to grow employees through employee involvement (Herrenkohl et al., 1999) [3].

Implementer's disposition/attitude is vital in building-worthy certificate policy execution. If implementers are well disposed towards a policy, they are more likely to carry it out as the original decision-makers intended. However, when implementers' attitudes or perspectives differ from the decision-makers, the process of implementing a policy becomes infinitely more complicated.

Commitment of the bureaucracy in realizing the vision and mission is the extent to which a Regional Government Work Unit leader, employees, or staff sides with a particular organization and realizes its goals or vision and mission, and intends to maintain its membership in the organization (Arfan Ikhsan et al., 2000). Mowday et al. (1979) state that commitment is “The strength of an individual's identification with and involvement in a particular organization.” Organizational commitment is a personal value that refers to a loyal attitude toward an organization.

Edward III in Widodo (2010: 104-105) [6] noted that for policy implementation to be effective and efficient, implementers must know what to do and have the will to do it.

Edward III in Widodo (2010: 100) says fiscal limitations and citizen opposition impede facility acquisition. The implementer's service quality is next. The low budget limits the quality of community services.

Bureaucratic support for building usage policies does not meet municipal rules, which require a building-worthy certificate. The Budget of the Public Works Department only budgets enough money for the Secretariat of the Building Expert Team (TABG) and the TABG Team Honor to operate. In the inspection phase of the building, an inspection tool is needed for reinforcing steel in concrete; it just has a hammer test and no infrared technology was used to observe concrete reinforcement. This needs buying equipment.

In addition to internal bureaucratic factors in the implementation of building policies for an operability certificate, there are external factors, notably, public support for these rules. In [12], the policy considers public participation in policy implementation. Implementation will run relatively more smoothly if the public is allowed to access the policy process, or at least in one of the processes such as setting an agenda or evaluating policies.

Public support for building legislation is explained in Government Regulation No. 36/2005 concerning the Implementation of Building Law No. 28/2002 Chapter V [13]. In the administration of buildings, community participation can play a role in monitoring and maintaining order, both in the activities of construction, utilization, preservation, and demolition of buildings. Monitoring is carried out objectively with full responsibility, with no disruption and/or loss for building owners and/or users, the community, and the environment. Based on its monitoring, the community reports in writing to the city government the indications of improper functioning of buildings and buildings whose construction, utilization, preservation, and demolition have the potential to cause disruption and/or danger to users, the community, and the environment.

Community support for the provision that buildings must have a Certificate of Appropriate Building Function before being occupied must be improved through public outreach and consultation. For residential function buildings, the community does not pay for building supervisors/technical service providers, but for business functions and select buildings, it does. Implementation of building-worthy certificates for building functions, notably the construction industry's willingness to work with the bureaucracy, is to examine building reliability.

Implementation of Article 68 of Regional Regulation No. 4 of 2008 Probolinggo City concerning Buildings [14] is that after 5 years, the existing buildings must have a building operability certificate that has not been fulfilled. Local regulation implementation is one of the stages of a public policy process that must be well planned. Mazmanian and Sabatier (1983: 4) say the foundation of policy implementation is understanding what should happen when a program is certified legal and basic policy in the form of local laws, implementing regulations, programs, and activities. During the 5 years of local regulation implementation, there were no mayor regulations, only the Building Expert Team Mayor Decree (TABG). The Building Function Eligibility Certificate Mayor's Regulation No. 13/2013 was issued in 2013, but not enforced until 2014.

Internal bureaucratic or Regional Apparatus Work Unit (SKPD) management factors (communication, bureaucratic structure, resources, and dispositions) and external factors of public support for building feasibility certificate policies on building functions must be resolved so that every building with a building permit also has the building operability certificate (SLF) so that it is safe to be inhabited and utilized.

The given description can generate the following research questions:

1. How does Regional Apparatus Work Unit (SKPD) implement the building operability certificate (SLF) in Probolinggo?
2. How can the building operability certificate (SLF) help Probolinggo achieve building-worthy performance certificates?

By problem formulation, this study's aims are as follows:

1. Review Technical Service building management to improve the building operability certificate
2. Review and analyze the implementation strategy of building operability certificate in achieving the goal of building function-worthy performance certificates in the city of Probolinggo

## 2. Literature Review

Based on the results of the discussion on the implementation, George Edward III suggests four implementation considerations. Communication, resources, disposition, and bureaucratic structure determine the success or failure of policy implementation (1980: 148). This model assumes that public policy implementers and policy performance are linear. According to Van Meter and Van Horn (Winarno, 2014: 159), six variables influence the implementation of policies: “(1) basic measures and policy objectives (standard and objectives of the policy); (2) resources of policy; (3) communication between organizations and implementation activities (Inter-Organizational Communication Enforcement Activities); (4) characteristics of the implementing agencies; (5) economic, social, and political conditions and (6) The disposition of Implementors” [15].

The use of the theory of the model of George C. Edward III (1980) and Van Horn and Van Meter (1975) in this article is intended that the implementation of the building operability certificate policy is influenced by internal factors, namely, communication, organizational structure, resources, and disposition, and external factors, namely, economic, social, and political conditions as well as the public, such as the financial strength of the building owner community in fulfilling the building operability certificate (SLF) requirements, namely, the as-built drawing requirements for buildings that do not yet exist and other conditions, the awareness of building owners in having the building operability certificate (SLF), and the legislature in supervising the performance of the city government in the field of SLF implementation.

Strategy formulation is carried out using gap analysis, general matrix strategy approach, Boston Consulting Groups (BSG) matrix, and SWOT matrix strategy. From this approach, what will be discussed in this study is a strategy with a SWOT matrix strategy formulation approach [16].

Sianipar and Entang in Management Analysis Techniques (2003: 68) say that the SWOT matrix can be used to develop several main strategies in the four interrelated quadrants and focus on the goals formulated according to the strengths of each agency.

The IE Matrix is a strategic management tool which is used to analyze the organization's position with the total IFAS score weighted as the *X*-axis and the total EFAS score weighted as the *Y*-axis. IE Matrix is divided into nine cells with the following provisions [17, 18]:

1. Cells I, II, and III describe the organization conditions as grow and build. Intensive, namely, market penetration, market development, and product development, or integrative, namely, forward, backward, and horizontal, can be more appropriate for these divisions.
2. Cells IV, V, and VI describe the organization conditions as hold and maintain. The strategies used are market penetration and product development.
3. Cells VII, VIII, and IX are the harvest and divest strategies. The strategies used are divestment, conglomerate diversification, and liquidation.

The stage of strategic priorities using the QSPM (Quantitative Strategic Planning Matrix) tool. According to David (2009), QSPM analysis guides organizations to objectively evaluate alternative strategies. The stages were carried out in conducting the QSPM [18].

Purnamasari H (2020) researched the effectiveness of public services by providing recommendations for function-worthy certificates (SLF) at the Banjarbaru City Housing and Settlement Service, where there are still convoluted service procedures that are difficult to understand by the public and a lack of awareness from employees of their duties and responsibilities as state servants and public servants to provide fair and equitable services.

The study shows that government employees deliver good services to the community, more people manage the building operability certificate (SLF), and operating costs are insufficient [19].

Zenith Navigati evaluated the Application of Functional Eligibility Certificates in Regional Regulation Number 1 of 2012 concerning Buildings in Malang City. The research results show that the application of the certificate of function of Malang City has yet to be maximized [20].

In the research on the Implementation of the Sidoarjo Regent's Regulation Policy Number 72 of 2017 concerning Procedures for Granting and Expansion of the building operability certificate (SLF), Octafialdo Shandy found that the implementation of the Sidoarjo Regent's regulatory policy is going quite well according to four implementation models and one implementation variable, but one implementation model has not gone well, namely, communication and resources. Information power is also included in the model so that business actors still need to fully obtain information on the procedures for granting and extending the Certificate of Building Function Worthiness [21].

The research of Octafialdo and Hartono determined the implementation of the Sidoarjo Regent's policy on granting and extending the Certificate of Building Functionality. The researchers used five informants and qualitative data analysis to support this research [22].

In this study, the building operability certificate (SLF) is discussed, namely, the certificate granted by the Regional Government to declare the feasibility of building functions before being able to be beneficial. However, the performance of the building management before construction is required to have a Building Approval (PBG) and the building operability certificate (SLF). One of the criteria for building performance in an area is the extent to which the building operability certificate (SLF) has been issued.

This research is expected to produce a strategy for the structure of the Technical Service, communication between Technical Service Units and the One-stop Investment and Licensing Service Office (DPMPTSP) that issues the building operability certificate (SLF) and improving human resources, and increasing human resources and the budget for procuring building inspection equipment to support the issuance of the building operability certificate (SLF).

## 3. Methodology

This study uses mixed methods with a survey approach. The descriptive qualitative/quasiqualitative method aims to make a systematic, factual, and accurate description of a social phenomenon or natural phenomenon [23]. Primary data collection is carried out through building institutions and secondary data collection is carried out through journals, websites, and city government institutional data. Alternative Strategy Questionnaire data are collected by using ten building policy decision officials as respondents.

The descriptive quantitative approach explains a phenomenon by using numbers describing its characteristics (QSPM).

First, qualitative data analysis utilizing SWOT determines the organization's control plan. Second, ranking strategies are analyzed to identify QSPM's priorities (David, 2011) [24].

## 4. Discussion

Of the building function-worthy certificate in improving the performance of the building function feasibility policy certificate and the results of in-depth interviews with key actors implementing the certificate policy strategies can be implemented to improve its performance. The strategy was prepared based on a SWOT analysis by looking at internal factors, namely, the strengths and weaknesses of the building implementing service, the strength factor of the office, which already has regional building regulations, bureaucratic structure, and SLF implementation, which has been implemented since 2008. The Service's weakness factor is the need for more communication with internal institutions and the external lack of human resources to handle SLF implementation and disposition. External factors based on opportunities, namely, legislative support, SLF as an insurance guarantee, plan to replace PBG, and Technical Service Awards for SLF implementation. While the external threat factor is the lack of public participation in owning the building operability certificate (SLF), SLF has yet to become the basis for building insurance guarantees, and the lack of solid professional teams of experts and individuals in implementing the building operability certificate (SLF).

Strategy determination was performed through the first stage of carrying out internal and external quantitative approaches to strategy analysis through the IFAS and EFAS scores. The second stage is conducting a SWOT analysis, and the third stage is prioritizing strategies by performing calculations by using a Quantitative Strategic Planning Matrix (QSPM) tool.

### 4.1. Internal and External Quantitative Approaches to Strategy Factor Analysis Summary

Feasibility of buildings according to the strengths, weakness, opportunities, and treatment matrices through a quantitative approach according to Tables 1 and 2of the qualitative approach SWOT through the SLF policy implementation strategy as follows [25, 26].

**Figure 1:**
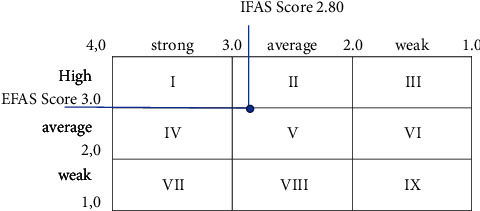
IE Matrix.

Figure 1 shows the organization's position (*x*; *y*) is (2.80; 3.00) in quadrant II. This indicates that SKPD subbuilding affairs can grow and develop. The strategies used in this quadrant are product or service development strategy or forward, backward, and horizontal integration strategy.

### 4.2. SWOT Strategy

The strategy for developing bureaucratic services forms the basis or strategy for forward, backward, and horizontal integration in the formulation of the strategy in Table 2.

### 4.3. Priority Strategy

Based on Table 2, SWOT strategy for implementing the building operability certificate (SLF) policy and the results of the choice of priority strategies for respondents, the priority for organizational development strategy with the following strategic priorities is shown in Table 3.

The QSPM tool is used in the determination of ranking strategies to obtain priority lists (David, 2011). By the results of the calculation of the Quantitative Strategic Planning Matrix (QSPM), the priority ranking of the implementation of the building operability certificate (SLF) policy and SWOT strategy for implementing SLF policy. In QSPM analysis, the value of attractive score (AS) is obtained from the opinion of the researcher. Strategies that have been obtained using SWOT analysis utilizing total attractive score (TAS) are obtained by multiplying AS and weight. The value of sum of total attractive scores (STAS) indicates the rank of the priorities. The results of QSPM analysis priority strategies based on the determination of policies from respondents are shown in Table 4 [25]:

From the priority results in Table 4, metrics of QSPM with the following strategy:

1. Improvement of the building operability certificate (SLF) implementation of the Laws and Regulations: Implementation of SLF is based on the latest laws and regulations following Law 11 of 2020 concerning Job Creation, Law 28 of 2002, and Government Regulation No. 16 of 2021 concerning Buildings and Adjustments to Regional Regulations of Probolinggo City No. 4 of 2008 concerning Buildings with Government regulations.
2. Increasing public awareness of the building operability certificate (SLF): Increased socialization of SLF management to the Building Owners' community
3. Strengthening the commitment of SKPD leaders in implementing the building operability certificate (SLF): SKPD leaders prioritize the implementation of SLF by instructing staff to prioritize the performance of SLF services starting from the completeness of data, technical recommendations, and issuance of SLF.
4. Renewal of SKPD's coordination in the inspection of building functions: Coordination was improved in the implementation of the building operability certificate (SLF), especially for the implementing agencies, namely, the coordination and communication of building services and investment and one-stop licensing services.
5. Increasing resources (human resources, budget, management information system (SIMBG), authority and facilities, and infrastructure) gradually.

Resources are increased by adjusting and expanding the building operability certificate (SLF) budget every year, starting from the human resource development skills of SIMBG service officers, the authority of SLF officers, and improving SLF facilities and infrastructure.

## 5. Conclusion

Based on the results of the discussion above, the following conclusions can be drawn:

1. First, highlighting the key factors that affect the implementation of building feasibility certificate policies, such as a bureaucratic structure that must focus on managing SLF implementation, does not take care of project implementation. No standard operating procedures (SOP) for a building-worthy certificate are regularly implemented, so they function according to the present conditions. Resources, namely, the budget lacking in SLF implementation, need to be added to fulfill the development of human resources, SIMBG facilities, and infrastructure. Communication needs to be improved by issuing SLF between the building office, the licensing services office, and the community that owns the building. And dispositions, namely, leadership and staff authority in implementing SLF, as well as social, economic, and political conditions needs to be improved by considering building owners' social and economic conditions in managing SLF and legislative support in implementing SLF.
2. Second, priority plan strategies will be implemented by building implementers in improving the implementation of building feasibility certificate policies, namely, (a) improvement of SLF implementation by the laws and regulations; (b) increasing public awareness of The SLF; (c) strengthening the commitment of SKPD leaders in implementing SLF; (d) renewal of SKPD's coordination in the inspection of building functions; and (e) increasing resources (human resources, budget, management information system (SIMBG), authority, and facilities and infrastructure) gradually.
3. Third, the challenge in the process of drafting building regulations that takes a long time is that the building administrator's SKPD must prepare building regulations by revising regional rules following government regulations, starting from coordination between institutions and legislative processes in the regions, provinces, and the central government and preparing regional head regulations regarding the implementation of SLF that are sufficient at the regional level. The performance of the SLF implementation strategy requires the readiness of resources (HR, budget, equipment, information systems, and authority) in stages before issuing the SLF. The increase in the limited SLF implementation budget each year is intended for developing human resources in SLF services, procurement of equipment skills of SMBG officers, and increasing the authority of SLF service officers. Intense communication between the building office and the licensing service is required in issuing the SLF.

## Figures and Tables

**Table 1:** Internal and external quantitative approaches to strategy factor analysis summary (IFAS and EFAS).

No.	Internal strategic factor	Weight	Weighting	Rating score
I	Strength			
	(1) There is a legal basis for BG regional regulations of buildings	0.19	5	0.95
	(2) Bureaucratic structure exists	0.18	4	0.72
	(3) The implementation of the building operability certificate (SLF) has been a long time	0.17	3	0.51

	Subtotal I	0.5		2.18

II	Weaknesses			
	(1) External communication is lacking	0.15	1	0.15
	(2) Resources (HR, funds, building information system, authority, and equipment) are lacking	0.16	2	0.32
	(3) Disposition/tendency of Regional Work Units (SKPD) leadership attitude is lacking	0.15	1	0.15

	Subtotal II	0.5		0.62

	Total weight (I + II); difference in weighting score (I−II) = *x*	1.0		2.80

	External strategic factors			

III	Opportunities			
	(1) Strong legislative oversight	0.17	4	0.68
	(2) As collateral for BG assets (insurance, ILO, hotel, and hospital certificates)	0.18	5	0.90
	(3) PKPD-PU Building Award	0.16	4	0.64

	Subtotal I	0.5		2.22

IV	Threats			
	(1) Public awareness (community and private) lack of care for SLF	0.17	2	0.34
	(2) SLF is not a mandatory requirement for bank guarantees (still IMB)	0.16	2	0.32
	(3) Technical assessors of individual BGs and agencies do not yet exist	0.16	2	0.32

	Subtotal II	0.5		0.98

	Total weight (I + II); difference in weighting score (I−II) =	1.00		3.00

Source: results of analysis, 2020. *Note.* Rating (Likert scale: 1–5); 5 indicates very high; 4 indicates high; 3 indicates quite high; 2 indicates lacking; and 1 indicates very less.

**Table 2:** SWOT strategy for implementing the building operability certificate (SLF) policy.

External bureaucratic factors	Internal bureaucratic factors	
	Strengths	Weakness (weakness)
	(1) The existence of a legal basis for building regulations, mayor regulations, and building laws (SI1)	(1) Lack of external communication (WI1)
	(2) Bureaucratic structure exists (SI2)	(2) Resources are lacking (WI2)
	(3) The implementation of SLF has been a long time (SI3)	(3) Disposition/tendency of SKPD leadership attitude is lacking (WI3)

Opportunities (opportunities)	SO strategy	WO strategy

(1) Strong legislative oversight (OE1)	(1) Improvement of SLF implementation based on local regulations, trustee, and PUPR regulation	(1) Increased communication optimally to building owners and construction service providers
(2) As collateral for BG assets (insurance, ILO, hotel, and hospital certificates) (OE2)	(2) Strengthening SKPD's SLF tasks and functions	(2) Gradually increasing resources
(3) PKPD-PU Building Award (OE3)	(3) Improving the implementation of SLF services every year	(3) Strengthening the commitment of SKPD leaders in implementing SLF

Threats (threat)	ST strategy	WT strategy

(1) Public awareness (community and private) lack of care for SLF (TE1)	(1) Increasing intensification of local regulations and building trustees	(1) Increasing public awareness about SLF
(2) SLF is not a mandatory requirement for bank guarantees (TE2)	(2) Renewal of SKPD's coordination in the inspection of building functions	(2) Increased resources in increasing SLF issuance
(3) Technical assessors of individual BGs and agencies do not yet exist (TE3)	(3) Facilitating the role of construction services in building SLF inspections	(3) The commitment of SKPD heads in empowering and increasing the capacity of construction service providers

Source: analysis results, 2020.

**Table 3:** Prioritas strategy (PS).

PS no.	Prioritas strategy
PS1	Improvement of the building operability certificate (SLF) implementation based on local regulations, trustee, and PUPR regulation
PS2	Increasing public awareness about building operability certificate (SLF)
PS3	Strengthening the commitment of SKPD leaders in implementing the building operability certificate (SLF)
PS4	Renewal of SKPD's coordination in the inspection of building functions
PS5	Increased resources in increasing the building operability certificate (SLF) issuance

Source: analysis results, 2020.

**Table 4:** QSPM.

Key factor	Weight	Alternative strategy									
		PS1		PS2		PS3		PS4		PS5	
		AS	TAS	AS	TAS	AS	TAS	AS	TAS	AS	TAS
*Strengths*											
SI1	0.19	4	0.76	3	0.14	2	0.38	3	0.57	2	0.38
SI2	0.18	3	0.54	3	0.10	3	0.54	3	0.54	3	0.54
SI3	0.17	4	0.68	4	0.13	3	0.54	2	0.36	2	0.36

*Weakness*											
WI1	0.15	3	0.45	4	0.07	3	0.45	4	0.60	3	0.45
WI2	0.16	4	0.64	3	0.10	4	0.64	4	0.64	4	0.64
WI3	0.15	3	0.45	3	0.07	3	0.45	3	0.45	3	0.45

*Opportunity*											
OE1	0.17	4	0.68	3	0.12	3	0.51	3	0.51	3	0.51
OE2	0.18	4	0.72	3	0.13	4	0.72	3	0.54	4	0.72
OE3	0.16	4	0.64	3	0.10	3	0.48	3	0.48	3	0.48

*Threatness*											
TE1	0.17	4	0.68	3	0.12	3	0.51	4	0.68	3	0.51
TE2	0.16	4	0.64	4	0.10	4	0.64	4	0.64	4	0.64
TE3	0.16	4	0.64	3	0.10	3	0.64	2	0.32	3	0.48

	STAS		7.56		6.38		6.340		6.33		6.16

	Priority		1		2		3		4		5

Source: analysis results, 2020.

## Data Availability

The data used in this study (Respondent Questionnaire Data for strategic priorities and strategic priority choices from respondents) are provided in the supplementary materials.

## Supplementary Materials

Supplementary Materials Supplementary File 1: –Respondent Questionnaire data for strategic priorities and strategic priority choices from respondents.
