# Supplementary material for: Strategies for Implementing Building Operability Certificate to Improve Performance of Building Management: A Case Study in Probolinggo City, Indonesia
Source: ScientificWorldJournal. 2024 Jan 6;2024:8749628. doi: 10.1155/2024/8749628 (PMC10787652; doi:10.1155/2024/8749628)
Supplement: Supplementary Materials — Supplementary File 1: –Respondent Questionnaire data for strategic priorities and strategic priority choices from respondents. [file 8749628.f1.pdf]

Alternative Strategy Questionnaire

No		Strategy SO												Strategy WO												Strategy ST												Strategy WT											
		SO1				SO2				SO3				WO1				WO2				WO3				ST1				ST2				ST3				WT1				WT2				WT3			
	Strengths Internal	1	2	3	4	1	2	3	4	1	2	3	4	1	2	3	4	1	2	3	4	1	2	3	4	1	2	3	4	1	2	3	4	1	2	3	4	1	2	3	4	1	2	3	4	1	2	3	4
1	The existence of a legal basis for building regulations, mayor regulations and building laws (SI1)																																																
2	Bureaucratic structure exists. (SI2)																																																
3	The implementation of SLF has been a long time. (SI3)																																																
	Weakness Internal																																																
1	Lack of external communication (WI1)																																																
2	Resources are lacking (WI2)																																																
3	Disposition/tendency of SKPD leadership attitude is lacking (WI3)																																																
	Opportunity External																																																
1	Strong legislative oversight (OE1)																																																
2	As collateral for BG Assets (insurance, ILO, Hotel and Hospital Certificates) (OE2)																																																
3	PKPD-PU Building Award (OE3)																																																
	Threaths External																																																
1	Public Awareness (community and private) Lack of care for SLF (TE1)																																																
2	SLF is not a mandatory requirement for bank guarantees (TE2)																																																
3	Technical assessors of individual BGs and agencies do not yet exist (TE3)																																																

Note: Please check the gray line marked "X" in number 1 = unattractive; 2 = somewhat attractive; 3 = quite interesting; 4 is very interesting.
Alternative Strategy :
SO1: Improvement of SLF implementation based on local regulations, trustee and PUPR women
SO2: Strengthening SKPD's SLF tasks and functions
SO3: Improving the implementation of SLF services every year
WO1: Increased communication optimally to building owners and construction service providers.
WO2: Gradually increase resources
WO3: Strengthening the commitment of SKPD Leaders in implementing SLF
ST1: Increasing intensification of local regulations and Building trustees
ST2: Renewal of SKPD's Coordination in the inspection of building functions
ST3: Facilitate the role of construction services in building SLF inspections
WT1: Increasing public awareness about SLF
WT2: Increased resources in increasing SLF issuance
WT3: The commitment of SKPD heads in empowering and increasing the capacity of construction service providers

STRATEGY PRIORITY SELECTION MATRIC WITH QSPM

No	Respondent's Name	SI1:SO1	SI2:SO2	SI1:SO3	WI1:WO1	W12:WO2	W13:WO3	OE1:ST1	OE2:ST2	OE3:ST3	TE1:WT1	TE2:WT2	TE2:WT2
1	Head of Public Works Department	4	4	3	3	3	3	3	4	4	4	4	3
2	Head of Creation Division	4	4	4	3	3	3	3	4	4	4	4	3
3	Head of the Building Section	4	3	4	3	3	4	3	4	3	4	4	3
4	Head of drinking water section	4	3	3	4	3	4	4	3	3	4	4	3
5	Head of Environmental Health section	4	2	3	4	4	4	4	3	2	3	4	4
6	Head of City Planning	4	2	3	2	4	4	4	3	2	3	3	4
7	Head of Space Control section	3	3	4	2	2	4	3	4	4	3	3	4
8	Head of Spatial Planning section	4	3	4	3	3	3	3	4	4	4	3	3
9	Head of Highways	4	4	3	3	3	4	3	3	3	4	3	3
10	Head of Road Construction section	3	4	2	3	4	3	3	3	2	4	2	2
	Jumlah	**38,00**	32,00	33,00	30,00	32,00	**36,00**	33,00	**35,00**	31,00	**37,00**	34,00	32,00
